# A Novel Classifier Based on Urinary Proteomics for Distinguishing Between Benign and Malignant Ovarian Tumors

**DOI:** 10.3389/fcell.2021.712196

**Published:** 2021-08-30

**Authors:** Maowei Ni, Jie Zhou, Zhihui Zhu, Jingtao Yuan, Wangang Gong, Jianqing Zhu, Zhiguo Zheng, Huajun Zhao

**Affiliations:** ^1^School of Pharmaceutical Sciences, Zhejiang Chinese Medical University, Hangzhou, China; ^2^The Cancer Hospital of the University of Chinese Academy of Sciences (Zhejiang Cancer Hospital), Hangzhou, China; ^3^Institute of Basic Medicine and Cancer (IBMC), Chinese Academy of Sciences, Hangzhou, China; ^4^Department of Physiology, Zhejiang Chinese Medical University, Hangzhou, China; ^5^Tongde Hospital of Zhejiang Province, Zhejiang Academy of Traditional Chinese Medicine, Hangzhou, China

**Keywords:** urinary proteomics, machine learning, ovarian cancer, non-invasive biomarkers, mass spectrometry

## Abstract

**Background:**

Preoperative differentiation of benign and malignant tumor types is critical for providing individualized treatment interventions to improve prognosis of patients with ovarian cancer. High-throughput proteomics analysis of urine samples was performed to identify reliable and non-invasive biomarkers that could effectively discriminate between the two ovarian tumor types.

**Methods:**

In total, 132 urine samples from 73 malignant and 59 benign cases of ovarian carcinoma were divided into C1 (training and test datasets) and C2 (validation dataset) cohorts. Mass spectrometry (MS) data of all samples were acquired in data-independent acquisition (DIA) mode with an Orbitrap mass spectrometer and analyzed using DIA-NN software. The generated classifier was trained with Random Forest algorithm from the training dataset and validated in the test and validation datasets. Serum CA125 and HE4 levels were additionally determined in all patients. Finally, classification accuracy of the classifier, serum CA125 and serum HE4 in all samples were evaluated and plotted via receiver operating characteristic (ROC) analysis.

**Results:**

In total, 2,199 proteins were quantified and 69 identified with differential expression in benign and malignant groups of the C1 cohort. A classifier incorporating five proteins (WFDC2, PTMA, PVRL4, FIBA, and PVRL2) was trained and validated in this study. Evaluation of the performance of the classifier revealed AUC values of 0.970 and 0.952 in the test and validation datasets, respectively. In all 132 patients, AUCs of 0.966, 0.947, and 0.979 were achieved with the classifier, serum CA125, and serum HE4, respectively. Among eight patients with early stage malignancy, 7, 6, and 4 were accurately diagnosed based on classifier, serum CA125, and serum HE4, respectively.

**Conclusion:**

The novel classifier incorporating a urinary protein panel presents a promising non-invasive diagnostic biomarker for classifying benign and malignant ovarian tumors.

## Introduction

Ovarian cancer (OC) is a common malignant disease and the fifth leading cause of cancer-related mortality in women (Siegel et al., 2021). The ovaries, located in the pelvic cavity, are relatively concealed. In addition, obvious clinical manifestations and effective diagnostic methods are lacking for early OC, making early diagnosis and discrimination from benign ovarian tumors difficult. In more than 70% cases, OC is diagnosed at an advanced phase (Dochez et al., 2019). The survival rates of OC have improved only slightly over the past few decades, and even in countries with abundant medical resources such as the United States and Canada, the 5-year survival rate remains around 47% after diagnosis (Lheureux et al., 2019).

Early stage or preoperative differentiation of benign and malignant tumors is critical to improve prognosis of patients with OC. Differentiation of malignant from benign tumors is recommended to facilitate referral of patients with malignant tumors to a specialized center or an oncology surgeon, since therapeutic results have been shown to be superior to general treatment by an obstetrician/gynecologist (Nagell and Miller, 2016; Abramowicz and Timmerman, 2017). Cancer antigen 125 (CA125) in serum is currently the most widely used tumor marker for detection of OC but has limited diagnostic specificity (Shipeng et al., 2019). Human epididymis protein 4 (HE4, also named WFDC2) in serum is another OC biomarker with better specificity than CA125 that has attracted significant research attention in recent years. However, HE4 levels may be affected by menopausal status and age (Cheng et al., 2020). Thus, clinical diagnosis of the two types is primarily conducted based on Risk of Malignancy Index, CA125/HE4, clinical symptoms, menopausal status and ultrasound imaging (Goff et al., 2004; Manegold-Brauer et al., 2014; Soletormos et al., 2016; Chacon et al., 2019). Clinically useful rules have been established by the International Ovarian Tumor Analysis group to distinguish between benign and malignant tumors. Nevertheless, in approximately 10–20% of cases, the nature of ovarian tumor remains undefined (Zhang et al., 2019). Therefore, novel effective methods and biomarkers for rapid, inexpensive and non-invasive monitoring of high-risk populations and preoperative discrimination between benign and malignant ovarian tumors are an urgent requirement.

As a readily available and cost-effective biospecimen, liquid samples provide a useful tool for cancer biomarker discovery. Serum is the most commonly used liquid biospecimen in clinical applications and scientific research. Urine is easily attainable with no requirement of an invasive procedure, making it more suitable for disease surveillance in high-risk patients requiring frequent examination. Additionally, proteins, peptides and metabolites excreted in urine are less complex and more stable than those in plasma, making urine a more suitable medium for biomarker discovery (Jing and Gao, 2018; Grayson et al., 2019). Urine has been routinely used as “non-invasive liquid biopsy” for clinical research and diagnosis (Thomas et al., 2016; Njoku et al., 2020; Zhao et al., 2020). To date, however, no urinary biomarkers have been identified that can effectively distinguish malignant from benign ovarian tumors.

Proteomics based on mass spectrometry (MS) is a powerful technique increasingly employed not only for high-throughput identification but also quantification of multiple proteins. Data-independent acquisition (DIA) MS has recently emerged as a promising alternative to data-dependent acquisition (DDA) for quantitative proteomics analysis (Azimzadeh et al., 2021; Prestagiacomo et al., 2021). The DIA technique is widely used in the context of multiplex biomarker detection from clinical specimens, such as plasma and urine (Fang et al., 2020; Burnap et al., 2021).

In this study, high-throughput urinary proteome analysis in DIA mode was applied for the discovery of urinary biomarkers. MS data were processed with DIN-NN software, which uses deep neural networks to distinguish real signals from noise, as well as new quantification and interference-correction strategies (Demichev et al., 2020). Machine learning strategy (Random Forest Algorithm) was subsequently applied to analyze the data matrix (training dataset) generated with DIA-NN software and establish a classifier for differentiating malignant from benign ovarian tumors. The classifier was finally validated in test and validation data sets. To our knowledge, this is the first study to effectively use a combination of DIA proteome analysis and machine learning strategy for OC biomarker discovery. The novel classifier should benefit auxiliary diagnosis and may be commercially developed into kits for effective non-invasive surveillance of high-risk populations.

## Materials and Methods

### Patients

Our study was approved by the Ethics Committee of Zhejiang Cancer Hospital and conducted according to the ethical guidelines of the Helsinki Declaration of 1964 and subsequent versions. Both benign and malignant tumors were histologically confirmed from biopsies and non-treated before patient enrollment. Patients with a history of neoplasm of any type and/or multiple neoplasms were excluded from this study. Pathological benign types mainly included mucinous cystadenoma, serous cystadenoma, and ovarian cysts while malignant OC types included high-grade serous carcinoma. Concentrations of serum CA125 and HE4 were detected using the electrochemiluminescence technique based on standard protocols. The cut-off value was 35 U/mL for CA125 and 140 pmol/L for HE4.

### Samples and Study Design

**FIGURE 1:**
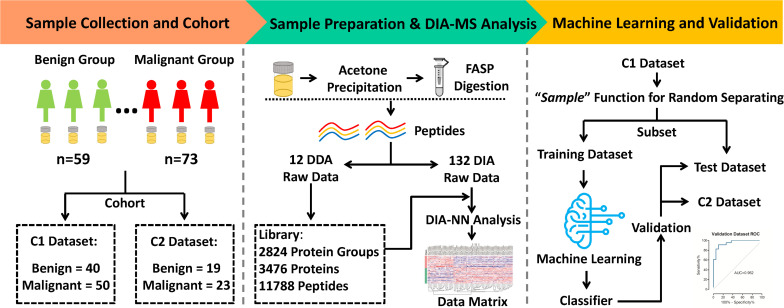
Workflow of MS analysis from urinary proteomics based on machine learning for distinguishing between benign and malignant ovarian tumors.

Morning midstream urine samples from 132 patients with ovarian tumors (including 73 malignant and 59 benign cases) were collected from 2018 to 2020. The clinical characteristics of patients are presented in Table 1. Two completely independent cohorts were set (Figure 1). The C1 dataset contained 40 benign and 50 malignant, while the C2 dataset contained 19 benign and 23 malignant samples. In the “Machine learning and Validation” platform, the C1 dataset was randomly divided into a training dataset (for machine learning to establish the classifier) and test dataset (for classifier validation) using the “*sample*” function in R software (version 3.6.1). As a completely independent cohort, the C2 dataset (also designated validation dataset) was used for further validation of the classifier.

**TABLE 1:** Clinical information of patients in this study.

	C1 Cohort		C2 Cohort
	Training dataset	Test dataset	Validation dataset
**Patients, number**			
Total	70	20	42
Benign	30	10	19
Malignant	40	10	23
**Age, year**			
Mean ± SD	54.0 ± 14.9	53.8 ± 13.4	53.8 ± 11.7
Median	55.5	54	54.5
Range	18–91	21–84	24–77
**BMI, kg/m^2^**			
Mean ± SD	22.5 ± 3.5	21.0 ± 2.1	22.2 ± 3.2
Median	22.4	21.3	22.4
Range	15.0–31.8	17.2–24.2	15.0–28.2
**Menopausal status, percentage**			
Menopause	62.9% (44/70)	60.0% (12/20)	59.5% (25/42)
Non-menopause	37.1% (26/70)	40.0% (8/20)	40.5% (17/42)

### Urine Sample Preparation

All urine samples were collected before treatment, divided into aliquots, immediately frozen and stored at −80°C. Urine samples were thawed on ice and centrifuged at 1,000 g for 5 min at 4°C to remove cell debris. Supernatant fractions were collected in new tubes. Cold acetone (supernatant: acetone, 1:4, v/v) was added to precipitate proteins overnight at −20°C. Samples were further centrifuged for 15,000 g for 15 min and protein pellets collected. Protein pellets were re-dissolved in lysis buffer (4% sodium dodecyl sulfate, 0.1 M Tris-HCl, pH 8.5) and protein concentrations assayed via bicinchoninic acid assay (Pierce, Thermo Scientific, Rockford).

Filter-aided sample preparation combined with sodium deoxycholate (SDC) was applied for protein digestion (Wisniewski et al., 2009; Erde et al., 2014). Briefly, 50 μg proteins was mixed with 200 μL of 8 M urea and transferred onto a filter device, followed by centrifugation at 15,000 g at 20°C for 15 min. The concentrate was washed with 8 M urea and centrifuged at 15,000 g for 15 min. After treatment with 0.1 M dithiothreitol and 0.05 M iodoacetamide, the concentrate was washed with 200 μL of 8 M urea and centrifuged twice at 15,000 g for 15 min. The concentrate was diluted with 100 μL of 50 mM ABC and centrifuged (this step was repeated twice) and subjected to trypsin digestion (enzyme to protein ratio 1:100, 50 mM ABC, 0.4% SDC) in a wet chamber at 37°C for 12 h. The digests were collected by centrifugation at 15,000 g for 15 min. Next, the filter device was rinsed with 50 μL of 0.5 M NaCl and centrifuged again. The resulting solutions were combined and acidified with 10% trifluoroacetic acid. Peptide solution was centrifuged at 14,000 g for 15 min and the supernatant collected into a new tube. Peptides were desalted using C18 tips (Pierce, Thermo Scientific, Rockford) according to the manufacturer’s instructions.

### DIA Library Construction

To generate a spectral library for analyzing DIA data from urine samples, peptides from all samples were collected into a single pool. The peptide pool was fractionated into 12 fractions using the Ultimate 3,000 UPLC system (Dionex, Idstein, Germany) coupled with an XBridge Peptide BEHC18 column (4.6 mm × 250 mm). Peptides were separated on a 75 min LC gradient at a flow rate of 0.5 mL/min. Mobile phase A comprised 2% acetonitrile (ACN) in water, pH 10.0, and mobile phase B contained 98% ACN, pH 10.0. The LC gradient was set as follows: 0–8 min, 100% A; 8–48 min, 100% A to 40% B; 48–53 min, 40% B to 100% B; 53–63 min, 100% B; 63–65 min, 100% B to 100% A; 65–75 min, 100% A. Peptides were eluted from 8 to 68 min. In total, 30 peptide fractions were collected, combined into 12 fractions and desalted as described above.

DDA data acquisition was conducted in a nano-LC & Q-Exactive system as reported previously (Ni et al., 2017). Desalted peptides were separated using an easy-nano LC system (Thermo Fisher Scientific, United States). The LC was connected to a 2 cm pre-column with an internal diameter of 75 μm filled with 5 μm C18 resin (Thermo Fisher Scientific). The pre-column was connected to a 25 cm analytical column with an internal diameter of 75 μm filled with 2 μm C18 resin (Thermo Fisher Scientific). The mobile component was composed of two phases: solution A (2% ACN/0.1% formic acid in water) and solution B (2% water/0.1% formic acid in ACN). Peptides were separated at a rate of 300 nL/min via stepwise-gradient elution: 0 min in 3% solution B, 10% solution B for 1 min, 25% solution B for 85 min, 30% solution B for 15 min, and 45% solution B for 2 min, followed by a column wash with 95% solution B for 17 min. MS spectra were acquired with Q-Exactive in a DDA mode, with automatic switching between MS and MS/MS scans using the Top 20 method. MS spectra were obtained at a resolution of 35,000 with an AGC target value of 3e6 or maximum injection time of 20 ms. Peptide fragmentation was performed via higher-energy collision dissociation with energy set at a normalized collision energy of 27. MS/MS spectra were acquired at a resolution of 17,500, with an AGC target value of 1e6 or maximum injection time of 60 ms, and the isolation window set at 2.0 m/z.

In total, we acquired 12 DDA files on a Q-Exactive in DDA mode. All DDA files were analyzed using the Proteome Discoverer (Version 1.4.1.14, Thermo Fisher Scientific) with Sequest HT search engine against a forward-decoy approach. The protein database composed of the *Homo sapiens* fasta database was downloaded from UniProtKB on 20 Jan 2020 containing 20,394 reviewed protein sequences. In total, the library contained 11,788 peptides and 2,824 protein groups.

### DIA-MS Analysis

The Nano-LC system and gradient for peptide separation were identical as described above (“DIA library construction”). Peptides eluted from the LC system were ionized at a potential + 2.0 kV into Q-Exactive mass spectrometer. A full MS scan was acquired (350–1250 m/z range) at a resolution of 35,000 (at m/z 200) in Orbitrap using an AGC target value of 3e6 and maximum injection time of 20 ms. Following the full MS scan, 33 MS/MS scans were acquired, each with a 17,500 resolution (at m/z 200), AGC target value of 1e6 and normalized collision energy of 27%, with the default charge state set to 2 and maximum injection time set to auto. The cycle of 33 MS/MS scans (center of isolation window) with a wide isolation window was as follows (m/z): 410, 430, 450, 470, 490, 510, 530, 550, 570, 590, 610, 630, 650, 670, 690, 710, 730, 750, 770, 790, 810, 830, 850, 870, 890, 910, 930, 950, 970, 990, 1,025, 1,075, and 1,125. DIA files were analyzed using DIA-NN software (v.1.6.0) with default parameters (Demichev et al., 2020).

### Quality Control of Mass Spectrometry and Methodology

For evaluation of the reproducibility of the MS platform, tryptic peptides of HeLa cell lysates were used as a quality control. A urine sample aliquot from each batch was processed as quality control of methodology reproducibility. Pearson correlation coefficient was calculated to evaluate the reproducibility of the platform and methodology with R v.3.6.1 using corrplot package.

### Statistical Analysis and Machine Learning

Proteins with > 30% missing ratios in C1 or C2 cohort were removed from the data matrix. Missing values of a particular protein were imputed with the minimum value of the protein in all samples. Log2 fold changes (log2 FC) in mean values of the comparison groups were calculated. Two-sided unpaired Welch’s *t-*test was performed for the comparison groups and adjusted *p*-values (also named *q*-value) calculated using a Benjamini & Hochberg correction. Significantly altered proteins were selected using the criteria of adjusted *p*-value < 0.01 and absolute log2 FC > 1. “Mean decrease in accuracy (MDA)” refers to a score reported by the “Random Forest” R package, which is used to evaluate the contribution of each feature to forest’s prediction accuracy. From the training cohort, we selected important protein features with MDA score > 5 using the random forest algorithm. In random forest analysis, 1,000 trees were generated using R package randomForest (version 4.6–14). Ten-fold cross validation was carried out with createFolds function in caret package and repeated 100 times. Five important features were selected for establishing the classifier, which was further validated in both test and validation data sets. Receiver operating characteristic (ROC) curves were calculated and plotted using pROC package (version 1.15.3). The Rtsne package was applied to plot t-SNE. The top Gene Ontology processes were enriched using a Metascape web-based platform (Zhou et al., 2019).

## Results

### Study Design and Quality Control

**FIGURE 2:**
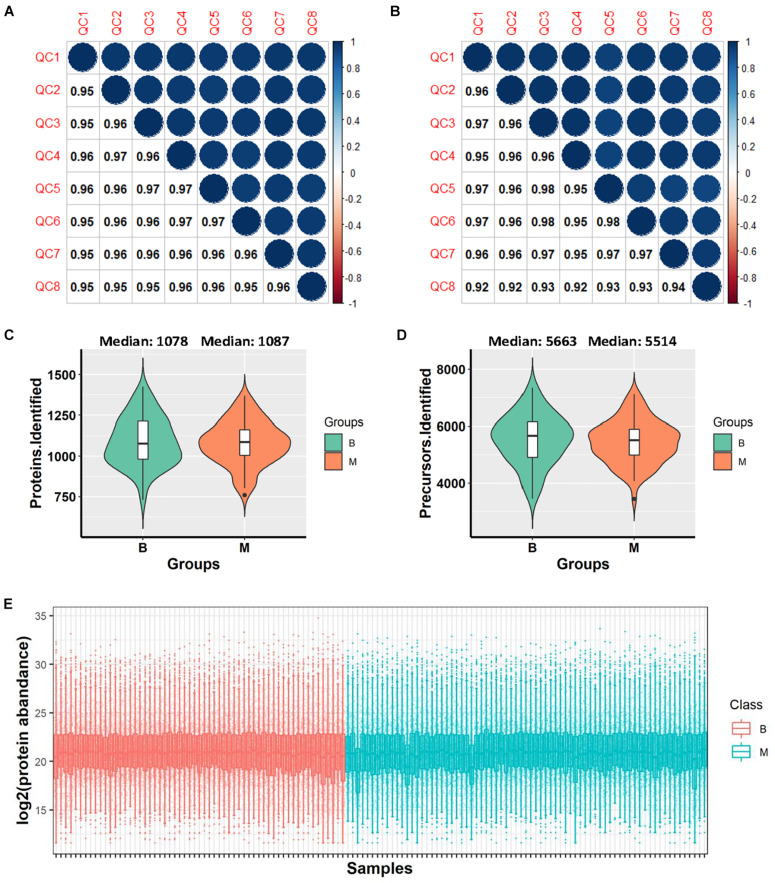
Data quality evaluation. **(A)** Pearson correlation of 8 HeLa cell lysate samples in all batches for evaluation of the reproducibility of mass spectrometry. **(B)** Pearson correlation of 8 repeat-tested urine samples in all batches for evaluation of the reproducibility of methodology. **(C)** Violin plot of protein identification numbers in benign and malignant groups. **(D)** Violin plot of precursor identification numbers in benign and malignant groups. **(E)** Box plot of protein abundance in each sample. *B, benign group; M, malignant group.

DIA-MS analysis was performed on urine samples from 132 patients. The samples comprised: (i) a discovery set C1 and (ii) an independent validation set C2 (Figure 1). The C1 dataset included urine samples from 40 benign and 50 malignant cases while the C2 dataset contained urine samples from 19 benign and 23 malignant cases. Samples were randomly distributed into 8 batches with the aid of 120 min DIA-MS, with quality control samples included in each batch. HeLa cell lysates and repeat-tested urine specimens were used as quality control samples to evaluate the reproducibility of the MS platform and methodology, respectively. The average Pearson Correlation Coefficient of protein quantitative data among HeLa cell lysates was 0.959 (range: 0.95–0.97, Figure 2A) while that among repeat-tested urine samples was 0.955 (range: 0.92–0.98, Figure 2B), supporting the consistent stability of both the MS platform and methodology. Median values of protein identification in the benign and malignant groups were 1,078 and 1,087 (Figure 2C) and median values of precursor identification were 5,663 and 5,514 (Figure 2D), respectively. The protein abundance profile of each sample in both groups was plotted, as shown in Figure 2E. The data quality of the two groups was consistently good.

### Proteomic Profiling of Benign and Malignant Groups

**FIGURE 3:**
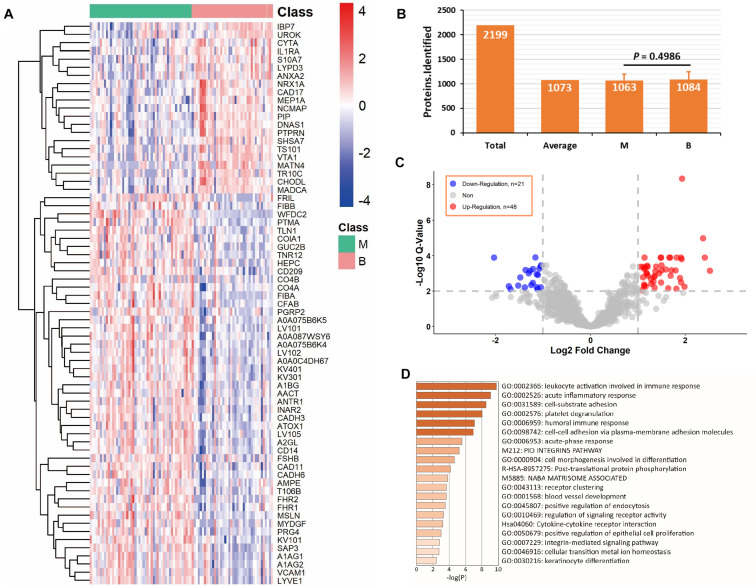
Analysis of differentially expressed proteins in C1 dataset. **(A)** Differentially expressed proteins in benign and malignant groups with *q*-value < 0.01 and absolute log2 fold change > 1. **(B)** Average protein identification numbers in each sample, benign and malignant groups of C1 dataset. **(C)** Volcano plot of down-regulation and up-regulation in malignant group. **(D)** Pathway analysis of the differentially expressed proteins using Metascape web-based platform. *B, benign group; M, malignant group.

The C1 dataset was used for proteomic profiling. Detailed patient descriptions in the dataset are presented in Table 1. In total, 90 samples, including 50 malignant and 40 benign samples, passed quality control in terms of protein identification (more than 500 proteins per sample). We identified and quantified 2,199 proteins and an average of 1,073 proteins was identified in each sample (Figure 3B). Overall, 1,063 proteins in the malignant group and 1,084 proteins in the benign group were identified, which were not significantly different in terms of number of proteins between the two groups (*P* = 0.4986, Figure 3B). We detected 69 differentially expressed proteins between the two groups (*q*-value < 0.01, absolute log2 FC > 1). Application of heatmap and volcano plot to differentially expressed proteins showed that 21 proteins were downregulated and 48 upregulated in the malignant group (Figures 3A,C). Pathway analysis of the 69 differentially expressed proteins revealed members of six major pathways, specifically, leukocyte activation involved in immune response, acute inflammatory response, cell-substrate adhesion, platelet degranulation, humoral immune response, and cell-cell adhesion (Figure 3D). These findings are consistent with the pattern of tumor progression. Clearly, compared with benign disease, malignant disease progression is commonly accompanied by alterations in the adhesion and migration abilities of tumor cells and a strong immune response (Gavalas et al., 2011; Boylan et al., 2016).

### Feature Selection and Classifier Development

**FIGURE 4:**
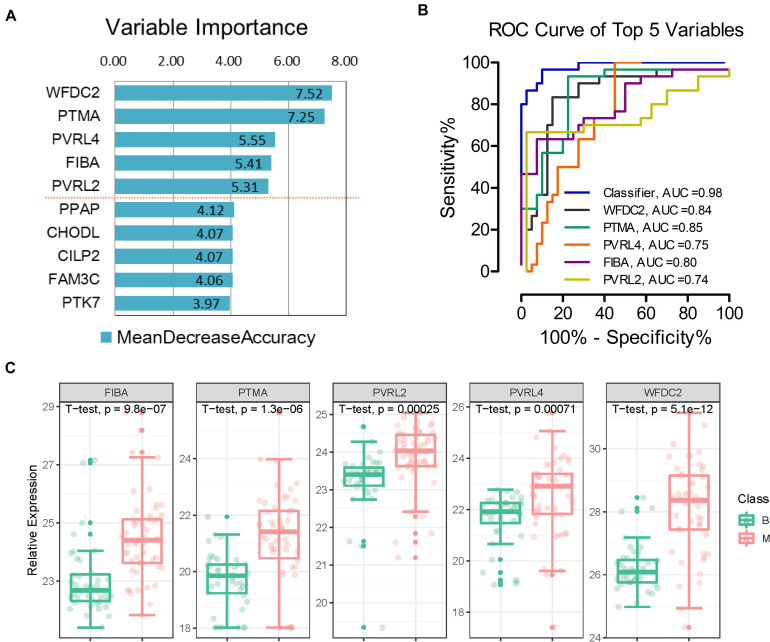
Separation of benign and malignant patients by machine learning of proteomic features. **(A)** Top 5 proteins prioritized by random forest analysis ranked by the mean decrease in accuracy > 5. **(B)** Receiver operating characteristic (ROC) analysis of the classifier and each feature in the training dataset. **(C)** Expression levels of the five proteins; *p*-value was calculated in *t*-test medtod. *B, benign group; M, malignant group.

**FIGURE 5:**
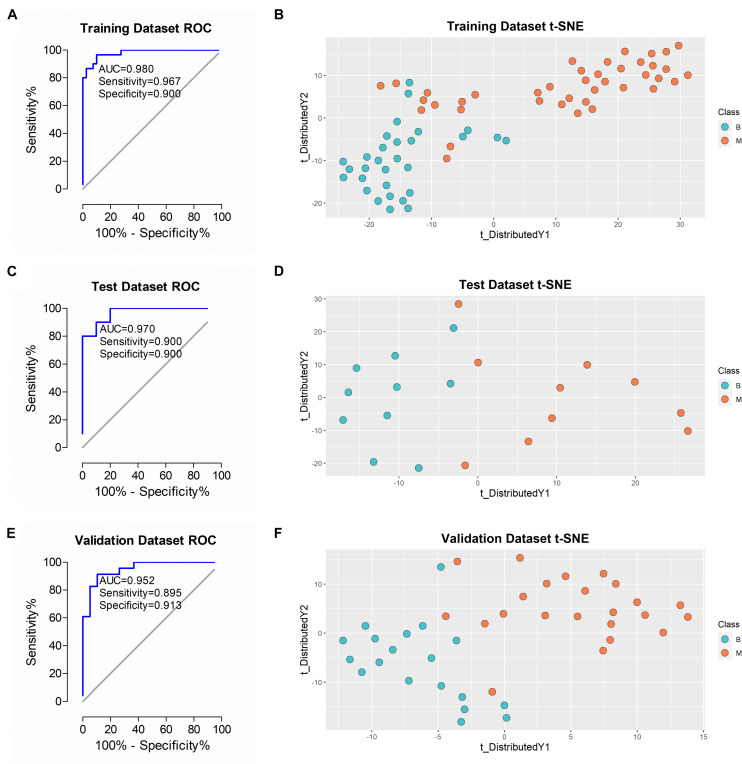
Performance of the classifier in diagnosing malignant from benign in different datasets. **(A,C,E)** ROC analysis of the classifier in training, test and validation datasets. **(B,D,F)** t-SNE analysis of the classifier in training, test and validation datasets. *B, benign group; M, malignant group.

To effectively identify potential biomarkers and conduct rigorous validation, the profiling dataset was randomly divided into training and test datasets (Figure 1). The training dataset (40 malignant and 30 benign samples) was used to screen potential features and construct classifiers for malignant diagnosis using random forest machine learning combined with 10-fold cross validation. The test dataset (10 malignant and 10 benign samples) was employed to validate the diagnostic effect of the classifier. We limited the number of selected features to facilitate practical evaluation using targeted proteomics or antibodies in the clinic. Using this approach, a classifier was established to distinguish between benign and malignant tumors, which contained five important variables (WFDC2, PTMA, PVRL4, FIBA, and PVRL2) with mean decrease in accuracy > 5 (Figure 4A). Expression levels of the five proteins across all 90 samples are presented in Figure 4C. Relative to the benign tumor group, these five proteins were significantly upregulated in the malignant tumor group (*P* < 0.05). Next, we calculated the area under curve (AUC) of the classifier in the training dataset. Furthermore, AUC values of the five features in the classifier were individually calculated. ROC plots showed that the classifier achieved an AUC value of 0.98 (Figure 4B and Figure 5A). Among the five features, AUC values ranged from 0.74 to 0.85, with the highest AUC of 0.85 obtained for PTMA (Figure 4B).

### Performance of the Classifier, Serum CA125 and HE4

After training and construction, the performance of the classifier was initially validated in the test dataset (*n* = 20 patients) comprising urine samples of 10 benign and 10 malignant cases. As shown in Figure 5C, ROC plot of the samples using the 5-protein classifier revealed AUC of 0.970. To ascertain accurate classification of the different sample types, we applied the t-SNE algorithm for visualization of performance. The t-SNE plot showed effective discrimination of malignant from benign samples in the test dataset (Figure 5D). The algorithm was additionally applied to visualize the performance of the classifier in the training dataset. Our results showed similar separation with some overlapping results (Figure 5B). To further validate this classifier in an independent patient cohort, 42 urine samples (C2 cohort) from 19 benign and 23 malignant cases were examined. To ensure rigorous validation, diagnoses were blinded during data acquisition and analyses. Each sample was analyzed using the identical DIA-MS workflow to the C1 cohort. Analysis of the resulting 42 DIA files led to the identification of an average of 1,107 proteins in each sample. AUC of 0.952 was achieved in a ROC plot of this dataset using the classifier (Figure 5E). The t-SNE plot clearly demonstrated effective differentiation between benign and malignant groups of ovarian tumors with our novel classifier (Figure 5F).

**FIGURE 6:**
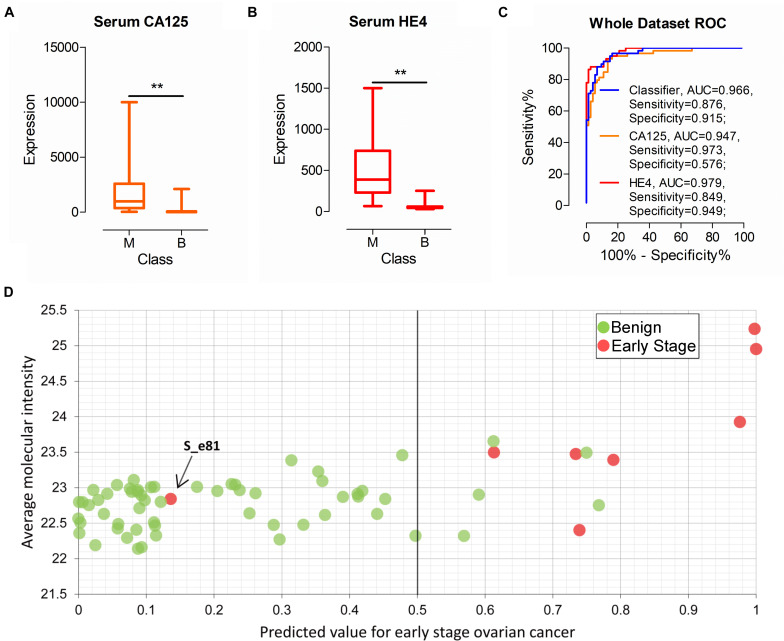
Performance of the classifier, serum CA125 and HE4 in all patients. **(A,B)** The expression of serum CA125 and HE4 in all patients. **(C)** ROC analysis of the classifier, serum CA125 and HE4 in all patients. **(D)** Performance of the classifier in early stage ovarian cancer diagnoses. **p-value < 0.01.

As commonly used clinical biomarkers for auxiliary diagnosis of OC, CA125, and H4 have attracted significant attention. Serum CA125 and HE4 levels of all patients (*n* = 132) were examined in this study. As shown in Figures 6A,B, CA125 and HE4 levels were significantly different between patients with malignant and benign disease, with median values of 975.00 vs. 25.00 (CA125, *P* < 0.01) and 386.95 vs. 51.96 (HE4, *P* < 0.01), respectively. Next, we evaluated the performance of the two biomarkers according to cut-off values of 35 U/mL (CA125) and 140 U/mL (HE4) used in the clinic. AUC of serum CA125 was 0.947 with sensitivity of 0.973 and specificity of 0.576 in all patients. AUC of serum HE4 was 0.979 with sensitivity of 0.849 and specificity of 0.949. Classifier performance, also evaluated in all patients, achieved AUC of 0.966 with sensitivity of 0.876 and specificity of 0.915 (Figure 6C).

Among all the urine samples, eight were diagnosed as early stage malignant cases (stage I or II) according to FIGO (International Federation of Gynecology and Obstetrics) stage. Out of the eight samples, seven samples were correctly identified as early stage by the classifier (Figure 6D). The performance of serum CA125 and HE4 on the eight samples was additionally evaluated. Overall, six and four of the eight patients were correctly classified using serum CA125 and HE4, respectively (data not shown). One patient sample, labeled S_e81, was incorrectly identified with the classifier (Figure 6D). The serum CA125 and HE4 levels of this patient were 34.5 U/mL and 89.2 U/mL, respectively, suggesting incorrect classification with both biomarkers based on the cut-off value used in clinic. Our collective findings indicate that early stage malignancy is similar to benign tumor types and may be indistinguishable based on biomarkers in body fluid.

## Discussion

OC is a major public health issue owing to its high concealment and low 5-year survival rates. Development of accurate early diagnosis techniques and differentiation of malignant from benign ovarian tumors have long been an important focus of research. While serum CA125 and HE4 are widely used for auxiliary diagnosis of OC in the clinic, non-invasive and accurate diagnostic methods or biomarkers are also important for surveillance of high-risk patients. The rapid development of MS techniques, in particular, DIA-MS, has facilitated identification and quantification of the urinary proteome (Muntel et al., 2015; Sajic et al., 2015). This study focused on machine learning to assist in non-invasive diagnosis of different ovarian tumor types based on DIA-MS analysis of the urinary proteome.

Over the past decade, the use of protein panels (classifiers) to improve accuracy of diagnosis has attracted significant research attention. Protein panels mined from MS data present a key factor in classifier construction. Machine learning outperforms conventional statistical methods owing to improved ability to identify variable, resulting in improved predictive performance (Roux-Dalvai et al., 2019; Captur et al., 2020; Shen et al., 2020), and thus broadly utilized to analyze data from numerous areas of biology, such as transcriptomics, metabolomics and proteomics. Machine learners include Bayesian classifiers, Decision trees, Rule-based learners, Support Vector Machines, Artificial Neural Networks, and Random Forest, all with specific advantages and disadvantages. In this study, we applied Random Forest to analyze large-scale proteomics data generated with DIA-MS owing to its efficiency on large datasets and ability to handle large numbers of attributes (Swan et al., 2013). Following construction of the classifier using a training dataset, we performed essential validation in other datasets to confirm its ability and accuracy. Based on the workflow of DIA-MS analysis, random forest machine learning, classifier construction and validation, a five-protein panel was finally mined and validated from the data matrix of urinary proteomics. Serum CA125, a widely used biomarker for diagnosis of OC, was only identified in a few samples in our study (data not shown), probably due to presence of low levels in urine. Thus urine CA125 failed the above statistical screening and was excluded from subsequent analyses. This invalidation of well-characterized serum biomarkers of OC, such as CA125, in urine suggests a distinct diagnostic system from that in serum (Zhao et al., 2020).

While a 5-protein panel (WFDC2, PTMA, PVRL4, FIBA, and PVRL2) was developed for classification of benign and malignant ovarian tumors for the first time in this study, all included proteins were previously reported to be associated with cancer, displaying dysregulated expression in serum, tissue or cell lines. WFDC2 (WAP four-disulfide core domain protein 2, also named HE4), a small secretory protein expressed in OC, is commonly used as a serum diagnostic biomarker (Heliström et al., 2003). In addition to numerous studies using blood as the biospecimen, the association between HE4 in urine and OC has been investigated (Jia et al., 2017). Our results are consistent with previous reports of higher concentrations of urinary HE4 in patients with malignant OC compared to benign ovarian tumors (Macuks et al., 2012; Liao et al., 2015). However, the urinary level of WFDC2 showed limited accuracy as a single biomarker with AUC of 0.84 in this study. Prothymosin alpha (PTMA) plays an important role in cell growth, proliferation and apoptosis (Moreira et al., 2013; Wang et al., 2017). Recent studies suggest that overexpression of PTMA is associated with tumorigenesis, tumor progression and prognosis in cancer (Zhang et al., 2014; Ha et al., 2015). Our experiments showed higher expression of PTMA in the malignant relative to the benign ovarian tumor group, supporting the tumor biomarker potential of PTMA. Accumulating evidence supports the utility of PTMA as a novel therapeutic target in several diseases, including cancer and inflammation (Samara et al., 2017; Zhu et al., 2019). FIBA (fibrinogen alpha), also designated fibrinogen alpha chain, is one of three polypeptide chains that make up the blood-borne glycoprotein fibrinogen. Comprehensive research has shown upregulation of serum FIBA in multiple cancer types (Duan et al., 2018; Shi et al., 2018). However, the utility of urinary FIBA as a biomarker of OC has not been established as yet. Experiments from the current study showed that the urinary FIBA level was significantly higher in patients with malignant than benign tumors. Combined with previous findings, our results support the potential of FIBA as a tumor biomarker (Duan et al., 2018; Shi et al., 2018). Recent findings suggest that both PVRL2 and PVRL4 (poliovirus receptor-related 2 and 4) are induced under cancer-promoting conditions and affect the functions of immune cells, such as T-cells and natural killer cells (Bekos et al., 2019; Whelan et al., 2019). Expression of PVRL4, also known as Nectin-4, on the surface of OC cells is reported to alter their adherence and migration ability (Boylan et al., 2016). We observed higher levels of urinary PVRL2 and PVRL4 in malignant tumor groups, indicating that overall expression of both molecules is positively correlated with OC.

Previous studies have shown that urine could enrichment changes in all parts of body and is a highly sensitive matrix indicative of pathological changes in the body (Wu and Gao, 2015). Thus, urine presents an early biomarker source with the potential to reflect small, early pathological changes signifying the onset of diseases such as cancer (Wu et al., 2020). Evaluation of the performance of our novel classifier supports its potential in non-invasive early stage diagnosis. Our future objective is to collect urine samples of patients with early stage malignant tumors on a large scale for validation of the early stage diagnostic capability of the classifier. If possible, a novel model using a combination of the classifier with serum CA125 or HE4 could be established to improve early stage diagnostic accuracy.

Although the classifier was established as an effective diagnostic marker with an achieved AUC of 0.952 in the validation dataset, several limitations of this study should be considered. First, our proteomic analysis does not allow absolute quantification. If the classifier is to be applied in the clinic or developed into a kit, more rigorous quantification and extensive validation analyses are warranted. Additionally, due to the relatively small sample size, we did not include more clinical parameters for machine learning analysis that could have further improved the diagnostic power of the classifier.

## Conclusion

In conclusion, DIA-MS based urinary proteomics was combined with machine learning to establish a novel classifier for discriminating between malignant and benign ovarian tumors in this study. Our collective results indicate that the newly established classifier presents a promising tool for non-invasive diagnosis of OC.

## Data Availability Statement

The datasets presented in this study can be found in online repositories. The names of the repository/repositories and accession number(s) can be found below: iProX database, accession no: IPX0003013000.

## Ethics Statement

The studies involving human participants were reviewed and approved by the Ethics Committee of Zhejiang Cancer Hospital. The patients/participants provided their written informed consent to participate in this study.

## Author Contributions

MN: funding acquisition, methodology, and writing—original draft. JZ: funding acquisition, and validation. ZhZ: software, and visualization. JY: supervision. WG: resources. JZ: investigation. ZgZ: data curation, investigation, and writing—review and editing. HZ: conceptualization, formal analysis, and project administration. All authors contributed to the article and approved the submitted version.

## Conflict of Interest

The authors declare that the research was conducted in the absence of any commercial or financial relationships that could be construed as a potential conflict of interest.

## Publisher’s Note

All claims expressed in this article are solely those of the authors and do not necessarily represent those of their affiliated organizations, or those of the publisher, the editors and the reviewers. Any product that may be evaluated in this article, or claim that may be made by its manufacturer, is not guaranteed or endorsed by the publisher.
